# From Prediction to Interaction: A Behavioral Informatics Framework for AI in Preventive Health

**DOI:** 10.2196/98961

**Published:** 2026-10-28

**Authors:** Anand Gavai, Jaap Heringa

**Affiliations:** 1 Bioinformatics Section, Department of Computer Science, Vrije Universiteit Amsterdam, De Boelelaan 1081A, Amsterdam, 1081 HV, The Netherlands, 0031 6 34 64 99 52

**Keywords:** artificial intelligence, AI, agentic AI, large language models, digital health, human-AI interaction, preventive health, behavioral informatics, digital behavior change

## Abstract

In this viewpoint, we present our perspective—informed by published evidence and by our own work on AI-driven digital health systems—on why AI has had limited real-world impact in preventive health and how it should be redesigned. Most AI systems in health care are built as episodic prediction tools, whereas preventive care depends on sustained behavioral change; the consequences are visible in consistently high attrition and low long-term adherence across digital health interventions. The aim of this viewpoint is to argue that this failure is structural rather than incremental and to propose an integrated, interaction-centric alternative. We advance 3 key messages. First, the limited impact of AI in preventive health reflects a structural misalignment between episodic, prediction-centric system design and the longitudinal dynamics of health behavior. Second, addressing this misalignment requires treating continuous interaction as the primary unit of design, which we articulate through a behavioral informatics framework integrating 6 interdependent layers: data, knowledge, reasoning, interaction, behavior, and governance. The contribution of this framework is explicitly integrative: it synthesizes previously separate strands, including just-in-time adaptive interventions, behavioral intervention technologies, and human-AI interaction research, into a single system-level account. Third, evaluation must shift from predictive accuracy and short-term engagement toward measurable behavioral trajectories and longitudinal outcomes, with explicit attention to feasibility and to conditions under which simpler, non-AI interventions remain preferable. This viewpoint is addressed to digital health researchers and AI developers, behavioral scientists, clinicians involved in preventive care, and policymakers responsible for governing continuously interacting AI systems.

## Introduction

### Episodic AI in a Longitudinal Domain

AI in health care has traditionally been structured around prediction, where systems transform input data into outputs such as risk scores, classifications, or recommendations. These model-centric approaches have demonstrated strong performance in domains such as diagnostics and decision support [1,2]. However, their impact in preventive health remains limited, because prevention depends not on isolated decisions but on sustained behavioral adaptation, where outcomes emerge from sequences of small, context-dependent actions repeated in everyday life [3,4].

The empirical record quantifies this gap. Eysenbach’s “law of attrition” identified steep nonusage attrition as a defining feature of eHealth interventions [5]. Subsequent studies found that only about 50% of participants fully adhered to web-based interventions [6], while app-based chronic disease interventions had a pooled dropout rate of 43% (95% CI 29%‐57%), rising to 49% in observational studies [7]. Across popular mental health apps, median retention was 3.9% at 15 days and 3.3% at 30 days [8]. Chatbot interventions have improved physical activity, diet, and sleep [9], but long-term maintenance remains uncertain. These findings, rather than predictive accuracy alone, motivate this viewpoint.

Generative AI and large language models (LLMs) enable personalized recommendations through conversational interfaces [10], but most implementations remain episodic: they respond to user input and terminate. Even conversational or agentic systems may not maintain longitudinal context or support sustained behavioral change [11,12]. Their implicit assumption—that accurate information is sufficient—is difficult to reconcile with persistent attrition. Information that is not engaged with cannot change behavior, and engagement is a central determinant of intervention effectiveness [13,14]. Episodic AI is therefore misaligned with the temporal dynamics of preventive health behavior.

### Definitions and Scope

Because several terms are used throughout this viewpoint, we define them at the outset. Interaction-centric AI denotes systems in which the primary unit of design and evaluation is the continuous loop of exchanges between system and user—insight, action, feedback, and adaptation—rather than the accuracy of individual outputs. A behavioral informatics framework is an integrated account that couples the informatics components of such systems (data, knowledge, and reasoning) with their behavioral components (interaction design and behavior change mechanisms) under explicit governance constraints. Agentic coordination refers to reasoning organized as the orchestration of multiple specialized components across user state, context, and knowledge, rather than autonomous decision-making by a single model [15].

We use preventive health in a deliberately broad sense that includes both primary prevention (interventions before disease onset) and secondary prevention or early risk management, such as lifestyle modification for individuals with elevated but subclinical risk factors, as in the prevention of hypertension, type 2 diabetes, and obesity. What unites these settings, and distinguishes them from acute or episodic care, is that outcomes depend on behavior sustained over months to years rather than on discrete clinical decisions.

### Aim, Contribution, and Roadmap

The aim of this viewpoint is to explain why episodic, prediction-centric AI is structurally unsuited to preventive health and to propose an integrated framework for designing and evaluating interaction-centric alternatives. Rather than restate the 3 key messages given in the abstract, we note where each is developed: the first in this Introduction and the following section, the second in the framework section and the illustrative scenario, and the third in the section on evaluation and feasibility.

Two additional points clarify the contribution and readership. Interaction-centric AI has been proposed previously, including in our work on agentic coordination [15] and human-AI interaction research [11,16]. This viewpoint contributes an integrative synthesis showing how behavioral, computational, and governance concerns couple. The framework sections primarily address AI developers and behavioral scientists, the feasibility subsection addresses clinicians and preventive care organizations, and the governance subsection addresses policymakers and regulators.

## From Episodic Prediction to Continuous Behavioral Support

### The Interaction Loop

Traditional health care AI uses linear pipelines that generate predictions or recommendations for users or clinicians to interpret and act upon [1,2]. This suits discrete decisions but not behaviors such as activity, diet, and sleep, which vary with routines, context, and motivation. A recommendation has little impact if it is not acted upon and reinforced; repeated context-specific actions can instead accumulate into meaningful outcomes.

Interaction-centric systems embed outputs in iterative cycles of insight, action, feedback, and adaptation. Insights are coupled with actionable guidance, behavioral responses are observed, and subsequent recommendations adapt. This requires longitudinal context, adaptation to changing user states, and calibrated interaction frequency, while introducing risks including fatigue and reduced autonomy.

### Positioning Relative to Existing Frameworks

Three research traditions have addressed parts of this problem, and our framework builds directly on them. Just-in-time adaptive interventions (JITAIs) formalize when to intervene: decision points, tailoring variables, and decision rules that deliver support at moments of receptivity [17,18]. The behavioral intervention technology (BIT) model formalizes what to deploy, organizing design around 5 questions: why the intervention exists (the clinical aim: eg, blood pressure reduction), what strategy serves it (eg, self-monitoring), how that strategy is instantiated conceptually (eg, a reminder), how it is delivered technically (eg, a push notification), and when delivery occurs (the workflow rule: eg, every evening at 8 PM) [19]. Human-AI interaction research formalizes how systems should communicate: guidelines for transparency, feedback, and user control at the interface [11,16].

The comparison is complementary. JITAIs use predefined decision points and rules, whereas interaction-centric systems may repeatedly derive decision points, strategies, and framing from evolving user state. BIT can also specify adaptive workflows in which delivery changes with predefined user or environmental variables. Here, adaptation is a recurring cross-layer operation that may revise decision points, behavioral strategy, framing, and delivery. For example, after observing that an 8 PM weekday reminder is ignored but weekend meal planning is engaged with, the system may shift to Saturday-morning action planning. The distinction is not static versus adaptive intervention, but whether runtime reconfiguration and its dependencies across data, knowledge, reasoning, behavior, interaction, and governance are central architectural processes. Governance also constrains each operating cycle rather than remaining solely external compliance. Existing traditions isolate timing, intervention content, or interface, yet failures often arise at their couplings: timing cannot rescue poor framing, framing cannot rescue context-insensitive advice, and failures remain invisible without behavioral feedback. A JITAI can therefore be one framework configuration with a fixed-rule reasoning layer, while BIT is a design specification that can be instantiated within the framework; the present architecture makes the cross-layer processes governing runtime adaptation explicit.

## A Behavioral Informatics Framework for Interaction-Centric AI

### Overview and Operating Cycle

We present a conceptual architecture of 6 interdependent layers—data, knowledge, reasoning, interaction, behavior, and governance (Figure 1)—that clarifies dependencies and design constraints rather than prescribing implementation. Continuous feedback couples the layers, and performance emerges from their interaction. Membership is determined by what each layer owns; explicit rules distinguish data from knowledge and interaction from behavior. All layers depend on a time-varying representation of user state, context, and behavioral trajectories.

A loop traversal proceeds as follows: (1) the data layer updates user state; (2) reasoning detects a decision point; (3) knowledge grounds candidate recommendations; (4) reasoning acts, defers, or remains silent and selects a strategy within governance constraints; (5) interaction chooses timing, framing, and modality; (6) behavior records the response and updates strategy estimates; and (7) the response re-enters the data layer. The following subsections describe each layer and its integration with care ecosystems.

**Figure 1.**
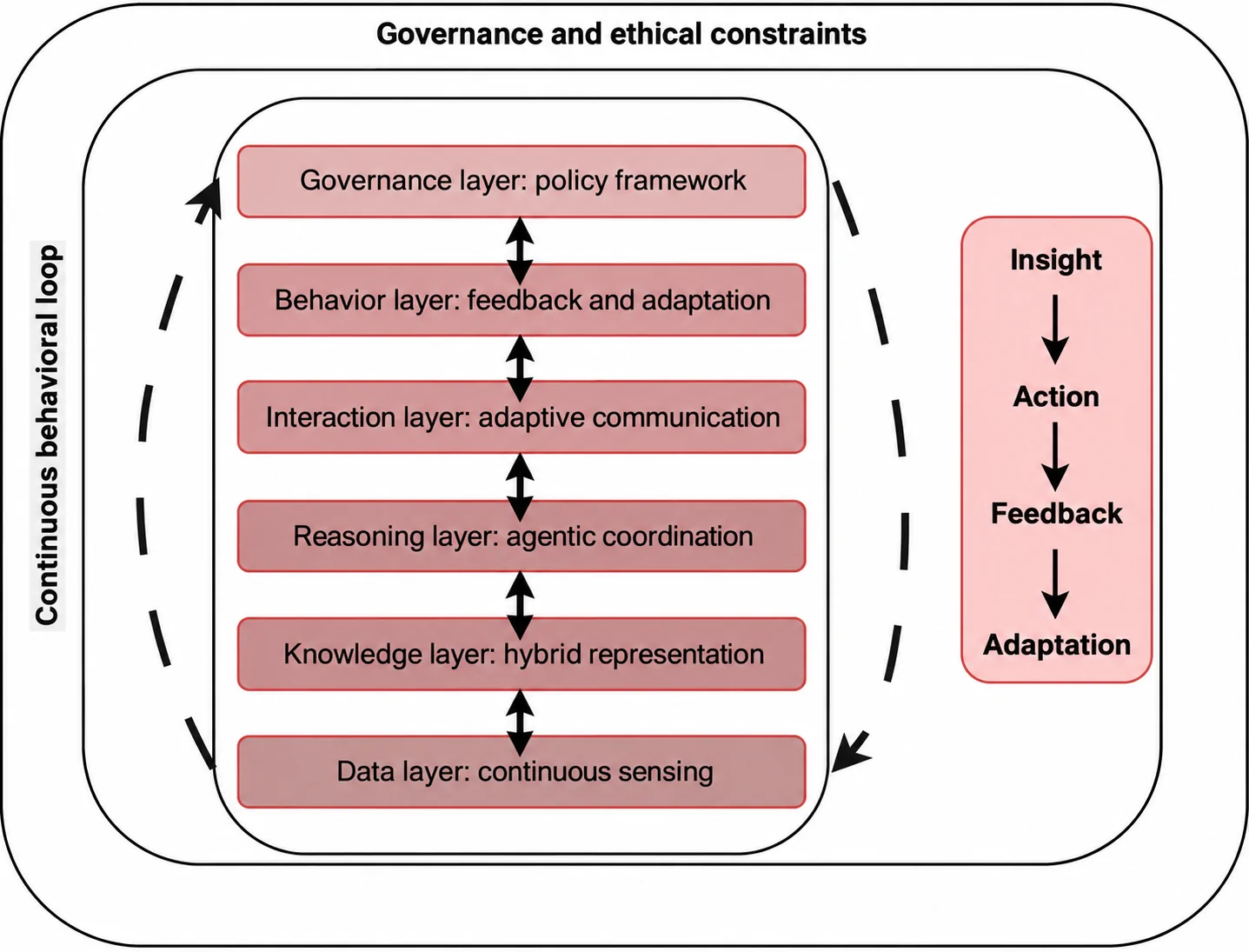
Conceptual architecture of an interaction-centric AI system for preventive health. Six interdependent layers connect continuous sensing, knowledge representation, reasoning, adaptive communication, behavioral feedback, and governance.

### Data Layer: Continuous and Contextual Inputs

The data layer owns what is true of this person—time-indexed observations and derived state—whereas the knowledge layer owns person-independent evidence. The distinction is not raw versus structured or sensed versus retrieved: a graph of the user’s history remains data, while a guideline threshold remains knowledge even when presented as retrieved text. The layers differ in update trigger (user action versus evidence change), validity condition (measurement reliability versus evidential currency), governance obligation (data minimization versus provenance), and failure mode (missensed steps versus outdated guidance). The reasoning layer joins them during decision formation. Data streams may include self-reports, sensors, and context. Although wearables and apps already collect many such data, they often lack integration with adaptive reasoning and behavioral feedback. Because longitudinal data are incomplete and noisy, this layer must construct a temporally coherent user state representation through signal integration, missing data handling, temporal smoothing, imputation, and adaptive sampling [20]. Collection must also limit burden and intrusion, which undermine engagement [17].

### Knowledge Layer: Hybrid Representation

Reliable recommendations require both retrieved sources such as clinical guidelines and structured representations such as knowledge graphs [12,21]. Retrieval accommodates evolving evidence; structure supports relational reasoning. Hybrid systems must resolve incomplete, outdated, or conflicting information [22] and maintain coherence with current evidence and prior behavior [23], escalating unresolved high-stakes conflicts to human oversight. Because sources are continuously updated and partly inconsistent, and LLM consistency over time remains contested [24], knowledge integration is an epistemic limitation as well as an engineering task.

### Reasoning Layer: Agentic Coordination

The reasoning layer integrates signals from the data and knowledge layers to generate context-aware, actionable outputs. Rather than relying on a single monolithic model, reasoning can be organized as coordination across specialized components for behavioral interpretation, contextual analysis, and knowledge grounding. In this framing, agentic AI means coordination across knowledge, context, and behavior, not autonomous decision-making, aligning with perspectives on AI as an orchestrator of distributed reasoning [15,25], and raising deliberate questions about how much autonomy is achievable or desirable in safety-critical health contexts.

Three functions define this layer. Personalization requires combining rule-based constraints, which guarantee safety and alignment with established evidence, with adaptive mechanisms responsive to individual trajectories, avoiding both rigidity and uncontrolled variability [26]. Temporal orchestration requires deciding not only what to recommend but when to intervene and when to remain passive [27]: excessive interaction produces fatigue, and insufficient interaction forfeits effectiveness [28]. Uncertainty handling requires representing and communicating confidence honestly because overconfidence undermines trust while excessive caution reduces usability [23]; situations exceeding the system’s competence must be identified and escalated to clinical care.

### Interaction Layer: Adaptive Communication

The interaction layer governs how decisions are communicated. Its scope is deliberately bounded: this layer owns the communication channel, timing, framing, modality, and dose of exchanges, whereas the selection of behavioral strategies and the monitoring of behavioral response belong to the behavior layer described next. The primary end user throughout this framework is the individual whose behavior is being supported, not the clinician; clinicians interact with these systems as secondary users through oversight and escalation pathways, where design requirements differ substantially (aggregated trends and provenance rather than progressive disclosure).

Communication design follows the principle of cognitive economy: each exchange delivers minimal, actionable information to limit cognitive load [29], with progressive disclosure offering concise guidance first and detail on request. Interactions must be nonintrusive; deferred under high cognitive load; prioritized when action is feasible [18]; and adapted to health literacy, preferences, and communication styles [10]. Such adaptivity carries a documented cost: simplified, tailored outputs can obscure the full basis of a recommendation, and LLM-generated guidance has been shown to deviate from clinical practice guidelines when operating on real-world, unstructured inputs [30], which is why explanation obligations and evidence grounding are assigned to this layer by the governance layer below.

### Behavior Layer: Behavior Change Mechanisms and Adaptation

Where the interaction layer determines how the system speaks, the behavior layer determines what the system is trying to change and whether change is occurring. It selects and adapts behavior change techniques—goal setting, self-monitoring, prompts and cues, feedback on performance, and reinforcement—in response to user state and context, consistent with established models of capability, opportunity, and motivation [3]. Goal setting and self-monitoring belong to this layer because they define and track behavioral targets; the interaction layer only renders them. The BIT model conceptualizes digital interventions as a mapping between clinical aims, behavioral strategies, and technological instantiation [19]; interaction-centric systems differ in continuously reconfiguring these mappings in response to observed behavior, which brings flexibility but also potential instability.

A taxonomy of techniques does not by itself address the dependency and autonomy concerns this viewpoint raises. Self-determination theory supplies the distinction that does: behavior may be regulated autonomously, through goals the person endorses, or through controlled regulation driven by external contingency, and autonomous regulation is generally more strongly associated with persistence once external support is withdrawn than controlled regulation [31]; meta-analytic evidence in health contexts links autonomy-supportive practice to psychological need satisfaction, autonomous motivation, and better health outcomes [32]. The architectural consequence is direct: the behavior layer’s objective is not adherence but the internalization of regulation, so a system that raises adherence while shifting regulation from autonomous to controlled has failed on its own terms. Persistence during and after tapering may therefore provide one observable indication that regulation has become more internalized.

Motivational interviewing supplies the corresponding communicative technology and strategies that are autonomy-supportive by construction: eliciting the person’s own reasons for change rather than supplying them, responding to ambivalence without confrontation, and keeping the decision to act the user’s [33]. Technology-delivered adaptations have been applied across chronic disease prevention and management, though reviews report wide variation in implementation fidelity [34], and systems delivering motivational interviewing through conversational AI are only now being characterized, with rigorous and standardized fidelity assessment remaining a major open problem [35]. The behavior layer’s strategy set should therefore be drawn from techniques compatible with autonomous regulation, and fidelity to them should be monitored rather than assumed.

This layer must detect disengagement, identify barriers to action, and adapt accordingly, modifying recommendations, adjusting interaction frequency, or reframing guidance. A central tension is maintaining engagement without fostering dependency: frequent prompts may raise short-term adherence while creating reliance on the system. Tapering strategies include prompt modulation, gradual reduction of intervention intensity, and transition to maintenance phases as behavioral patterns stabilize; these strategies support the shift from guided interaction to autonomous behavior. Interventions optimized for adherence can shade into subtle behavioral influence, a risk taken up under governance.

This locates a boundary the viewpoint has so far only named: when does behavioral support become manipulation? Persuasive technology research defines persuasion as influence that changes attitudes or behavior without coercion or deception, and catalogs the system features through which it operates [36,37], but it assumes a strategy fixed at design time and in principle inspectable; an interaction-centric system reconfigures its strategy at run time, so what the user is subject to is neither fixed nor visible. Accounts of online manipulation supply the criterion that survives this shift: influence is manipulative when it exploits cognitive or affective vulnerabilities and depends on the target not recognizing it [38]. A substantial reduction in an adaptation’s effectiveness when its purpose and mechanism are disclosed may therefore serve as a warning sign of manipulative influence, although disclosure sensitivity alone is not sufficient to establish manipulation. The behavior layer should therefore record which strategy is active and why, while the interaction and governance layers should make that strategy inspectable and contestable by the user. Users should be able to request an explanation of the strategy’s purpose and operation.

### Governance Layer: Constraints and Accountability

Because these systems continuously influence behavior, governance is an architectural layer and design driver. The General Data Protection Regulation and the European Union Artificial Intelligence Act principles—data minimization, purpose limitation, transparency, and human oversight—must be operationalized [39,40]. Adaptive sampling and on-device processing can minimize collection; behavioral data must not silently become training data for other purposes. The knowledge layer requires provenance and conflict resolution audit trails; the interaction layer requires explanations at recommendation time [41]; reasoning requires escalation thresholds; and the behavior layer requires user-controlled prompt limits, inspectable and contestable goals, and penalty-free disengagement.

Equity is a governance concern of the same rank: sustained engagement is achieved more readily among digitally literate and motivated users, so without explicit safeguards—inclusive communication design, multimodal interaction, attention to access constraints—interaction-centric systems risk amplifying existing health inequities. Finally, governance must address system-level risks of prolonged interaction, including bias, drift, and behavioral influence that is difficult to monitor when personalization is opaque; continuous auditing and monitoring are required to keep systems aligned with user needs and societal expectations [15]. Choices about data collection, personalization, and interaction strategy are normative: they embed assumptions about acceptable influence, autonomy, and risk.

### Ecosystem Integration

Interaction-centric systems must integrate with electronic health records, wearables, and other applications while addressing interoperability and cross-system governance [42]. Hybrid human-AI pathways should enable clinicians to review outputs, monitor trends, and intervene beyond automated scope [11,16]. Deployment must cover onboarding, active engagement, maintenance, scalability, robustness, and behavioral or performance drift [43]. Table 1 summarizes the layers, challenges, and evaluation metrics.

**Table 1.** Core layers of interaction-centric AI systems and associated design considerations and evaluation metrics^a^.

Layer	Primary function	Example capabilities	Key design challenges	Evaluation metrics
Data	Continuous and contextual input	Behavior tracking, sensor integration, and user-reported data	Missing data, noise, temporal drift, and user burden	Data completeness, signal reliability, sampling frequency adherence, and user input compliance
Knowledge	Context grounding and reasoning support	Hybrid representations (retrieval and structured knowledge)	Inconsistency, outdated information, and conflict resolution	Retrieval relevance, factual consistency, knowledge coverage, and update latency
Reasoning	Coordination across inputs and knowledge	Personalized recommendations and adaptive decision logic	Coherence, uncertainty handling, and control vs flexibility	Recommendation consistency, uncertainty calibration^b^, decision accuracy (contextual), and adaptation effectiveness
Interaction	Communication with the user	Adaptive prompts, progressive disclosure, and multimodal interaction	Cognitive load, engagement vs fatigue, and usability variability	Interaction quality (user ratings), response time, drop-off rate, and cognitive load indicators^b^
Behavior	Feedback and behavioral adaptation	Behavioral loops, reinforcement strategies, and habit formation support	Sustained adherence, behavioral variability, and disengagement	Behavioral adherence, action completion rate, habit persistence, and longitudinal engagement
Governance	Ethical, regulatory, and safety constraints	Transparency, explainability, user control, and compliance mechanisms	Trust, bias, regulatory compliance, and accountability	Explainability scores^b^, user trust measures, audit outcomes, bias metrics, and compliance adherence

a The evaluation metrics presented in this table, including behavioral adherence, action completion rate, and drop-off rate, can be measured using existing instruments, except for those identified by superscript b.

b Uncertainty calibration, cognitive load indicators, and explainability scores require further operationalization and context-specific validation for longitudinal interaction-centric AI systems.

The structural coherence of the framework does not guarantee practical viability. Interaction-centric systems presuppose sustained engagement, reliable behavioral signals, and consistent adaptation—conditions rarely met in real-world settings, as the attrition evidence cited above makes clear. There is a real risk that such systems reproduce the failures of earlier digital health interventions under a new architectural label, particularly if behavioral loops degrade into intermittent or superficial interaction. We treat this as a testable failure mode rather than a footnote, and return to it under feasibility below.

## The Framework in Operation: An Illustrative Scenario

To demonstrate how the layers interact in practice, consider an individual identified through routine screening as having elevated blood pressure, managed initially through lifestyle modification without pharmacological treatment. This is a secondary prevention setting—early risk management before established disease—and thus falls within the scope of preventive health defined in the Introduction. The scenario is a speculative design walkthrough, not a validated result; we annotate each step with the layer it exercises.

The system identifies dietary, activity, and routine patterns from wearable signals and meal logging (data). Elevated evening sodium intake combined with prolonged sedentary periods creates a decision point (reasoning), grounded in guidelines and user context (knowledge). At meal planning, it suggests a low-sodium alternative and prompts a brief walk (interaction). Logging and sensor data show whether the user acts (behavior); the system reinforces effective patterns or adjusts timing and framing, such as moving suggestions to weekend meal preparation. Governance caps interaction frequency and data sampling, while sustained blood pressure elevation triggers clinical escalation.

Over successive loop traversals, the system tapers from frequent intervention to an adaptive monitoring role, providing targeted input only when deviations from stable patterns are detected. Each element of this walkthrough corresponds to one step of the operating cycle described above; the scenario is the cycle instantiated in a concrete preventive context.

## Evaluation, Feasibility, and Implications

### From Predictive Accuracy to Measurable Behavioral Trajectories

The shift to interaction-centric AI requires rethinking evaluation. We propose 3 dimensions, each with measurable indicators. Interaction quality covers the relevance, timing, and usability of system outputs: measurable now through prompt response rates, time-to-response, conversation or session drop-off rates, and validated usability and engagement instruments [14,43]. Behavioral adherence covers whether interactions translate into action: measurable as the proportion of recommended actions completed within defined time windows (sensor-verified where possible), consistency of targeted routines, and time-to-disengagement, analyzed with survival methods. Longitudinal impact covers health-relevant change: trajectories of risk factors (eg, blood pressure, weight, and glycemic markers) and validated behavioral instruments, analyzed as slopes and inflection points in mixed-effects or growth-curve models rather than as single end point differences. Additional longitudinal indicators include time to habit formation, persistence of change after intervention tapering, and relapse rates.

Evaluation data arise largely from interaction traces, sensors, and periodic validated self-report. Individual adaptation complicates between-arm comparisons and supports within-person randomization. Microrandomized trials randomize intervention options at decision points, estimating proximal effects where the framework locates its mechanism [44]. Unlike standard person-level designs, their estimand is the effect of interaction policies rather than a fixed package. Conventional randomized controlled trials remain necessary for distal health outcomes, and AI-supported trial design is emerging [45].

Evaluation dimensions interact, and not always benignly. Increased adherence may come at the expense of autonomy, and optimized interaction frequency may accelerate fatigue. Adherence itself is not neutral: it presupposes goals that may not reflect user priorities, and systems optimized for adherence risk shifting from support toward subtle control, particularly when adaptation mechanisms are opaque or difficult to contest. Evaluation of interaction-centric systems is therefore normative as well as technical, and the boundary between support and manipulation must be an explicit evaluation criterion rather than an afterthought. Because evaluation data are continuously produced, they should feed back into system operation—a closed-loop process in which evaluation contributes directly to system evolution.

### Feasibility, Resources, and When Simpler Interventions Suffice

Feasibility remains uncertain. Persistent attrition [5,7,8] means no architecture guarantees continued engagement. Continuous sensing burdens devices and users; LLM reasoning creates recurring costs; escalation requires clinical capacity; and health-record integration demands maintenance. These constraints define where interaction-centric systems can justify their complexity.

Simpler interventions may be more cost-effective for motivated users with short-term goals, low-risk populations, or single barriers such as forgetting. A stepped-care approach should begin with the simplest adequate intervention and escalate only when relapse, multifactor risk, or failed alternatives justify added complexity. Head-to-head and noninferiority comparisons with simple alternatives should therefore be standard; superiority over inexpensive baselines is an empirical obligation, not an assumption.

## Conclusions

This viewpoint has argued that the limited real-world impact of AI in preventive health is structural: episodic, prediction-centric systems are misaligned with a domain whose outcomes emerge from behavior sustained over time. The behavioral informatics framework presented here reorients design around continuous interaction, integrating data, knowledge, reasoning, interaction, behavior, and governance into a single system-level account whose effectiveness depends on the couplings between layers rather than on any component in isolation. Interaction-centric systems must balance engagement against autonomy, personalization against comparability, and adaptability against stability, while operating within ethical and regulatory constraints.

Evaluating such systems requires metrics centered on behavioral trajectories and longitudinal outcomes rather than predictive accuracy alone, supported by designs such as microrandomized trials that match the unit of randomization to the unit of intervention. It remains an open question whether this level of system complexity is warranted in all preventive contexts; simpler interventions will sometimes achieve comparable outcomes at lower cost and risk, and continuous interaction carries its own hazards of overmonitoring, dependency, and cognitive burden. Future work should translate these principles into real-world implementations, validate them across diverse populations, and develop shared evaluation frameworks. Achieving this will require collaboration across AI, behavioral science, health care, and human-computer interaction. AI in preventive health is best understood not as a predictive tool but as an ongoing sociotechnical process embedded in everyday behavior.

## Abbreviations

BIT: behavioral intervention technology
JITAI: just-in-time adaptive intervention
LLM: large language model
